# Simulated microgravity facilitates cell migration and neuroprotection after bone marrow stromal cell transplantation in spinal cord injury

**DOI:** 10.1186/scrt184

**Published:** 2013-04-01

**Authors:** Takafumi Mitsuhara, Masaaki Takeda, Satoshi Yamaguchi, Tomotaka Manabe, Masaya Matsumoto, Yumi Kawahara, Louis Yuge, Kaoru Kurisu

**Affiliations:** 1 Department of Neurosurgery, Graduate School of Biomedical and Health Sciences, Hiroshima University, 1-2-3 Kasumi, Hiroshima, Minami-ku 734-8551, Japan; 2 Division of Bio-Environmental Adaptation Sciences, Graduate School of Biomedical and Health Sciences, Hiroshima University, Hiroshima, Japan

**Keywords:** Bone marrow stromal cell, Migration, Simulated microgravity, Spinal cord injury, Survival, Trophic factor

## Abstract

**Introduction:**

Recently, cell-based therapy has gained significant attention for the treatment of central nervous system diseases. Although bone marrow stromal cells (BMSCs) are considered to have good engraftment potential, challenges due to *in vitro* culturing, such as a decline in their functional potency, have been reported. Here, we investigated the efficacy of rat BMSCs (rBMSCs) cultured under simulated microgravity conditions, for transplantation into a rat model of spinal cord injury (SCI).

**Methods:**

rBMSCs were cultured under two different conditions: standard gravity (1G) and simulated microgravity attained by using the 3D-clinostat. After 7 days of culture, the rBMSCs were analyzed morphologically, with RT-PCR and immunostaining, and were used for grafting. Adult rats were used for constructing SCI models by using a weight-dropping method and were grouped into three experimental groups for comparison. rBMSCs cultured under 1 *g* and simulated microgravity were transplanted intravenously immediately after SCI. We evaluated the hindlimb functional improvement for 3 weeks. Tissue repair after SCI was examined by calculating the cavity area ratio and immunohistochemistry.

**Results:**

rBMSCs cultured under simulated microgravity expressed *Oct-4* and *CXCR4*, in contrast to those cultured under 1 *g* conditions. Therefore, rBMSCs cultured under simulated microgravity were considered to be in an undifferentiated state and thus to possess high migration ability. After transplantation, grafted rBMSCs cultured under microgravity exhibited greater survival at the periphery of the lesion, and the motor functions of the rats that received these grafts improved significantly compared with the rats that received rBMSCs cultured in 1 *g*. In addition, rBMSCs cultured under microgravity were thought to have greater trophic effects on reestablishment and survival of host spinal neural tissues because cavity formations were reduced, and apoptosis-inhibiting factor expression was high at the periphery of the SCI lesion.

**Conclusions:**

Here we show that transplantation of rBMSCs cultured under simulated microgravity facilitates functional recovery from SCI rather than those cultured under 1 *g* conditions.

## Introduction

In the last decade, a variety of cell types, including human neural stem cells [1], embryonic stem (ES) cell derivatives [2,3], and adult bone marrow stromal cells (BMSCs) [4-6], have been transplanted into the injured spinal cord of rats or mice, to their neuroregenerative activities. These preclinical studies showed that engrafted stem cells promote substantial functional recovery after spinal cord injury (SCI) through both cell-autonomous/cell-replacement and paracrine/trophic effects [7]. BMSCs are suggested to have the capacity to differentiate into neural lineages [8] and are advantageous with regard to less-invasive harvest procedures and large cell yields. BMSCs are also clinically attractive because they lend themselves to autologous transplantation in humans. Neural-like cells derived from BMSCs could be used in cell therapy for degenerative or traumatic diseases of the central nerve system [9-11]; thus, the efficacy of these cells as transplants has been investigated for the treatment of SCI [4,6,12,13].

We have focused on culturing BMSCs under simulated microgravity and reported its influence on important cellular events, such as cell cycling, proliferation, and differentiation [14-17]. Previous studies using human mesenchymal stem cells and human hematopoietic progenitor cells have indicated that simulated microgravity suppresses cellular differentiation, while favoring cell proliferation [16,18]. Our previous study showed that culturing of mouse BMSCs under simulated microgravity enhances the survival percentage, through maintenance of an undifferentiated state of pluripotent cells [17]. Moreover, in a mouse model of cerebral contusion, the efficacy of grafting mice BMSCs into the damaged brain appeared to be attributed not only to cells differentiating into neuronal cells but also to the factors derived by the grafted cells. These factors suppressed the formation of a glial scar and enhanced the elongation of the axons. In particular, mice BMSCs cultured under simulated microgravity expressed CXCR4 on their cell membrane; this expression was increased over cells grown under standard 1 *g* conditions, and the motor function of mice receiving grafts of BMSCs cultured under simulated microgravity improved significantly. These advantages make BMSCs cultured under simulated microgravity a strong candidate for cell-transplantation therapy for SCI.

In this study, we investigated the morphologic changes and trophic effects of rat BMSCs cultured under simulated microgravity on neural migration and survival, and on functional improvements after SCI in a rat model.

## Materials and methods

All study protocols were approved by the Animal Testing Committee Guidelines at Hiroshima University and/or the regulations of Animal Testing Facility of the Hiroshima University Natural Science Support Center. Animal care and handling procedures were in accordance with National Institutes of Health guidelines.

### Preparation of rBMSCs

Bone marrow cells of 5-week-old female Fischer/F344 rats were obtained from the bilateral femoral and tibial bones, and 1.0 × 10^7^ cells were suspended in Dulbecco modified Eagle medium, low glucose (Sigma-Aldrich Co., Saint Louis, MO, USA), with 10% fetal bovine serum (Thermo Fisher Scientific HyClone, South Logan, UT, USA), penicillin (100 units/ml), and streptomycin (100 μg/ml; both from Sigma-Aldrich) in 90-mm-diameter culture dishes (Thermo Fisher Scientific Nunc A/S, Roskilde, Denmark). Cells were maintained at 37°C with 5% CO_2_ in a humidified chamber, and the medium was exchanged to eliminate floating cells after 48 hours. Rat BMSCs (rBMSCs) adhering to the bottom of the culture dish were used as culture cells.

To obtain a sufficient population of cells for the experiment, two subcultures of rBMSCs were proliferated and plated by using OptiCell (Thermo Fisher Scientific Nunc A/S) at a density of 2.0 × 10^4^ cells/cm^2^. At 70% confluency, the cells were divided (day 0) and cultured under two different conditions: standard gravity (group 1G) and microgravity attained using the 3D-clinostat (group CL). After 7 days of culture, the rBMSCs were analyzed morphologically, with RT-PCR and immunostaining, and were used for grafting.

### 3D-clinostat

Microgravity conditions can be produced either by space flight or by free fall; to simulate microgravity, we used a 3D-clinostat (Mitsubishi Heavy Industries, Ltd., Kobe, Japan), as previously patented (undifferentiated pluripotent stem cell proliferation/differentiation regulation method and system, patent 2001–197182, Japanese published unexamined application 2003–9852, Foreign patent WO2004/061092 A1 PCT; US, EU, 2004). This device produces an environment similar to that of outer space (10^-3^*g*) by rotating a sample around two axes, integrating the gravity vector with the temporal axis. This is accomplished by rotation of a chamber at the center of the device, resulting in uniform dispersion of the gravity vector within a spherical volume, with a constant angular velocity. These specific conditions produced a simulated environment of 10^-3^*g* in 10 minutes.

### Morphologic changes

Morphologic changes of the cells were examined by using an inverted phase-contrast microscope (Eclipse TE 300; Nikon Co., Tokyo, Japan), and were recorded by taking images at random.

### RT-PCR

Cultured cells were collected by using ISOGEN (Nippon Gene Co., Ltd., Toyama, Japan), and RNA was isolated according to the manufacturer’s protocol. Reverse transcription was performed with ReverTra Ace-α- (Toyobo Co. Ltd., Osaka, Japan). By using cDNA as the template, PCR was performed by using BD Advantage 2 PCR Kits (BD Biosciences Clontech, Palo Alto, CA, USA). We used *Oct-4* as a pluripotency marker and CXC-chemokine receptor 4 (*CXCR4*) as the cell-migration marker. We also investigated the expression of neurotrophins (nerve growth factor (*NGF*) and brain-derived neurotrophic factor (*BDNF*)). Glyceraldehyde-3-phosphate dehydrogenase (*G3PDH*) was used as a housekeeping gene. The sequences of the primers as well as the PCR conditions used are shown in Additional file 1: Table S1.

### Immunostaining of cell-migration marker

Fixed rBMSCs were stained by using an immunostaining method and were examined by using a multifunctional microscope (BZ-9000; KEYENCE Co., Osaka, Japan). For CXCR4, a 400-fold dilution of monoclonal anti-CXCR4 (fusin; Santa Cruz Biotechnology, Santa Cruz, CA, USA) was used as the primary antibody, followed by Alexa Fluor 488 goat anti-mouse IgG (H + L) (Invitrogen Co., Carlsbad, CA, USA). For nuclear staining, a 500-fold dilution of 4^′^,6-diamidine-2-phenylindole dihydrochloride (DAPI; Kirkegaard & Perry Laboratories, Gaithersburg, MD, USA) was used. Images were stored on a computer for later analysis, and the percentage of positivity was calculated by dividing the number of positive cells by the total number of cells.

### Construction of a rat model of spinal contusion and cell grafting

Adult female Fischer/F344 rats weighing 150 to 200 g were purchased from Charles River Breeding Company (Yokohama, Japan) and were used for constructing a spinal-contusion model by using a weight-dropping method [13,19,20]. Rats were anesthetized, and a midline linear incision was made over the T9 to T11 spinous processes. By dissecting the bilateral paraspinal muscle laterally, the laminae of T9 to T11 were exposed. Laminectomy was carried out at T10. A cylindrical brass weight (10 g) was dropped down a stationary rod onto an impactor rod that rested on surface of the T12 dorsal dura mater. Spinal contusion was achieved with a force of 50 *g*/cm. The rats that underwent SCI received forced exercises for motor recovery of hindlimbs daily after the surgical procedure. Prophylactic antibiotics were administered for 7 days after the surgical procedure, and the bladders of these rats were expressed manually twice daily until spontaneous urine voiding was achieved.

### Experimental groups and cell transplantation

Rats were grouped into three experimental groups for comparison in our study; Group Control, in which PBS was administered immediately intravenously after the spinal contusion; Group 1G, which received intravenous injection of rBMSCs of group 1G cells immediately after spinal contusion; and Group CL, which received intravenous injection of rBMSCs of group CL cells immediately after spinal contusion.

Rats in groups CL and 1G received cellular grafting by injection of 3.0 × 10^5^ cultured cells diluted in 100 μl PBS into a tail vein. For the evaluation of survival and migration of BMSCs *in vitro*, the grafted rBMSCs were labeled by using PKH-26 (Sigma-Aldrich) as per established protocols [21]. Immunosuppressants were not used in any animals.

### Behavioral analysis

The Basso-Beattie-Bresnahan locomotor rating scale (BBB scale) [19,22] and inclined-plane task score were used to evaluate the hindlimb functional improvement of treated animals with spinal cord contusion. The BBB scale is a 22-point scale that systematically and logically follows recovery of hindlimb function, and ranges from a score of 0, indicative of no observed hindlimb movements, to a score of 21, representative of a normal ambulating rodent [23]. The inclined-plane task score assesses the maximum angle at which the animal could maintain its position for 5 seconds on an inclined plane. In this study, behavioral analysis was performed just before spinal injury, again every day from day 0 to 7, day 14, and day 21 after injury. All evaluations of motor function were performed by an observer lacking knowledge of group identities.

### Histologic and immunohistochemical analysis

At 3 weeks after transplantation, animals were deeply anesthetized and transcardially perfused with 4% paraformaldehyde for histologic and immunohistochemical analyses. The injured spinal cords were freed from the vertebral columns and cryoprotected in 30% sucrose. Fixed spinal cords were embedded in Tissue-Tek O.C.T compound (Sakura Finetechnical Co., Tokyo, Japan), frozen in liquid nitrogen, and sliced coronally into 7-μm-thick sections by using a cryostat (Leica Microsystems GmbH, Wetzlar, Germany). The segments were mounted on microscope slides to be used for hematoxylin and eosin (H&E) staining, staining of neurofilament heavy chain (NF-H) as a neural differentiation marker, and staining of glial fibrillary acidic protein (GFAP) as a glial differentiation marker. H&E-stained segments were examined under a multifunctional microscope (BZ-9000; KEYENCE Co.), and the areas of injured cavities were measured in group Control, group 1G, and group CL, by using digital image-processing software (ImageJ; National Institutes of Health, Bethesda, MD, USA). The cavity area ratio of each group was calculated by dividing the cavity area by the total coronally resected spinal cord area.

The following primary antibodies were used: monoclonal anti-neurofilament 200 (Sigma-Aldrich) for NF-H and monoclonal anti-GFAP (Sigma-Aldrich) for GFAP. Alexa Fluor 488 goat anti-mouse IgG (H+L; Invitrogen) was used as the secondary antibody. Stained segments were examined under a multifunctional microscope (BZ-9000). Transplanted PKH-26 labeled cells were also observed under this microscope.

Immunostaining of apoptosis-associated markers was performed for the apoptosis-promoting marker Bcl-2-associated X protein (Bax), and for the apoptosis-inhibiting markers B-cell CLL/lymphoma 2 (Bcl-2) and survivin. The following primary antibodies were used: anti-Bax monoclonal antibody for Bax, anti-Bcl-2 polyclonal antibody for Bcl-2, and anti-survivin polyclonal antibody (all; Santa Cruz Biotechnology, Santa Cruz, CA, USA). Secondary antibodies included enzyme-conjugated anti-mouse Bax monoclonal antibody for Bax, enzyme-conjugated anti-rabbit Bcl-2 polyclonal antibody for Bcl-2, and enzyme-conjugated anti-rabbit survivin polyclonal antibody for survivin (all, Santa Cruz Biotechnology). Mayer hematoxylin was used to stain nuclei. Stained segments were examined under a multifunctional microscope (BZ-9000), and the positivity ratios calculated by dividing the number of positive cells by the total number of cells.

### Statistical analysis

All data are expressed as mean ± SD of individual samples per group and were analyzed by one-way analysis of variance by using the Statistical Package for Social Sciences 19 software package for Windows (SPSS, Chicago, IL, USA). Differences between the values of two groups were assessed by using the Mann–Whitney *U* test. A *P* value of <0.05 was considered statistically significant.

## Results

### Effects of simulated microgravity for rBMSCs

**Figure 1:**
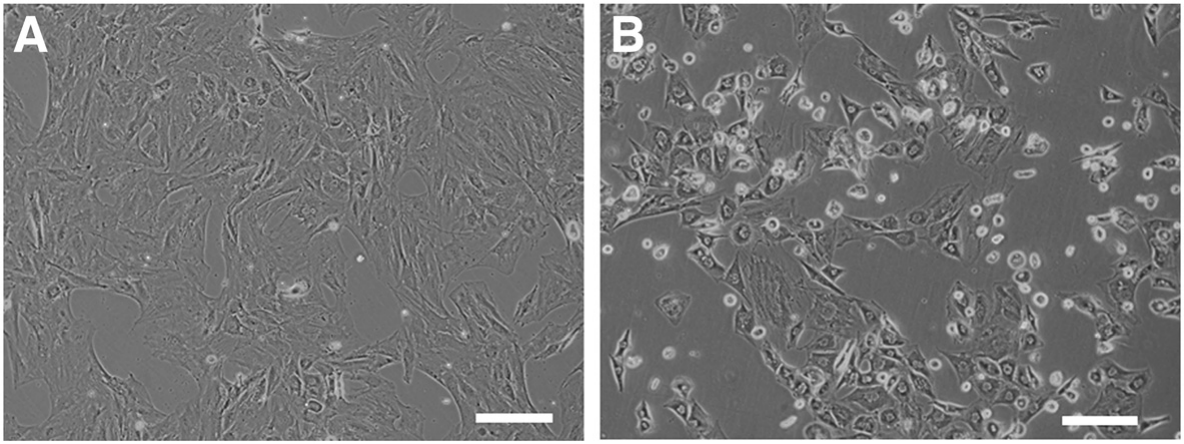
**Morphologic changes of each group.** Morphologic changes (**A**) group 1G; (**B**) group CL). The cells cultured under simulated microgravity (group CL) were changed to dome-like shape and were smaller than those cultured under 1 *g* (group 1G). Scale bars, 100 μm.

**Figure 2:**
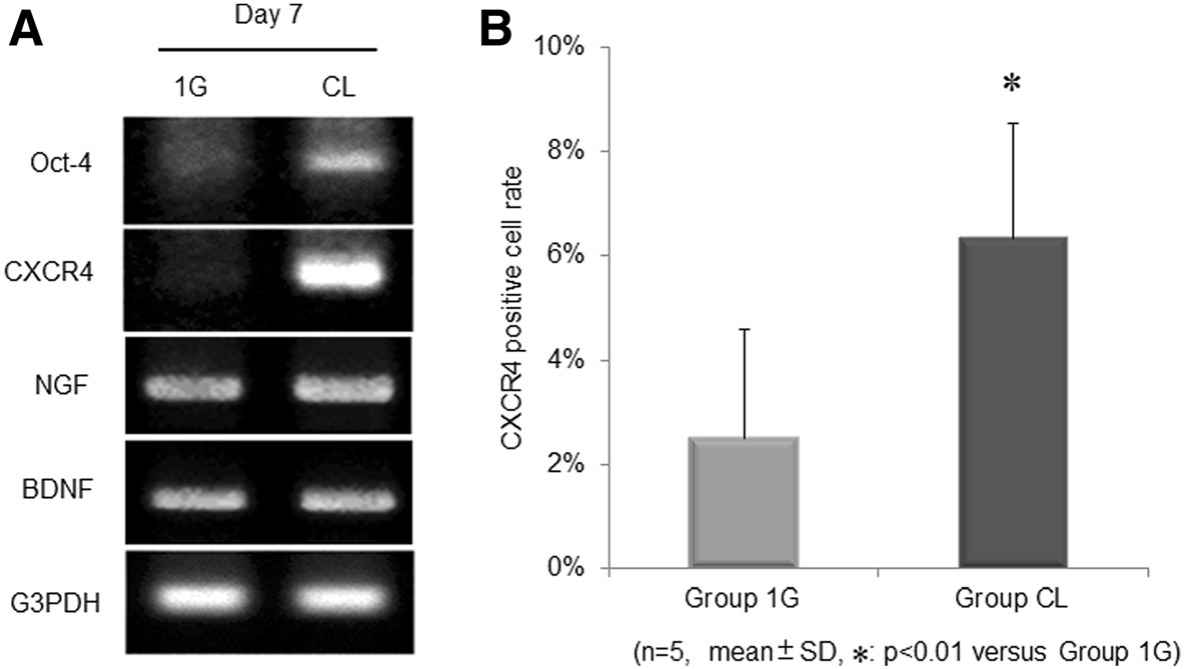
**mRNA expressions of the graft cells.** Effect of simulated microgravity on the graft cells. (**A**) mRNA expressions of *Oct-4*, *CXCR4*, *NGF*, and *BDNF* at day 7. The expression of *Oct-4* and *CXCR4* mRNAs was observed to be stronger in group CL cells. Expression of the housekeeping gene *G3PDH* was used to standardize expression. Immunostaining (**B**) indicated that the number of CXCR4-positive cells was significantly greater (*P* < 0.01) in group CL than in group 1G (*n* = 5 per group). Data are expressed as mean ± SD. CXCR4-positive cell percentage was significantly higher (*P* value < 0.01) in group CL than in group 1G.

Cells of both groups showed a spindle and oval shape, but the rBMSC morphology cultured under simulated microgravity was changed to “dome like” shape, and the cells were smaller than those cultured under 1 *g* (Figure 1A, B) (See Additional file 2: Figure S1: morphologic change of rBMSCs under simulated microgravity in detail). On day 7, expression of *Oct-4* and *CXCR4* mRNAs was observed to be stronger in group CL cells (Figure 2A). No difference was found between groups 1G and CL with regard to the expression of *NGF* and *BDNF* mRNAs. The number of cells positively stained for CXCR4 was significantly higher (*P* < 0.01) in group CL cells than in group 1G cells (Figure 2B).

### Effects of cell transplantation in SCI model rats: recovery of motor function, cavity repair, and migration of transplanted rBMSCs

**Figure 3:**
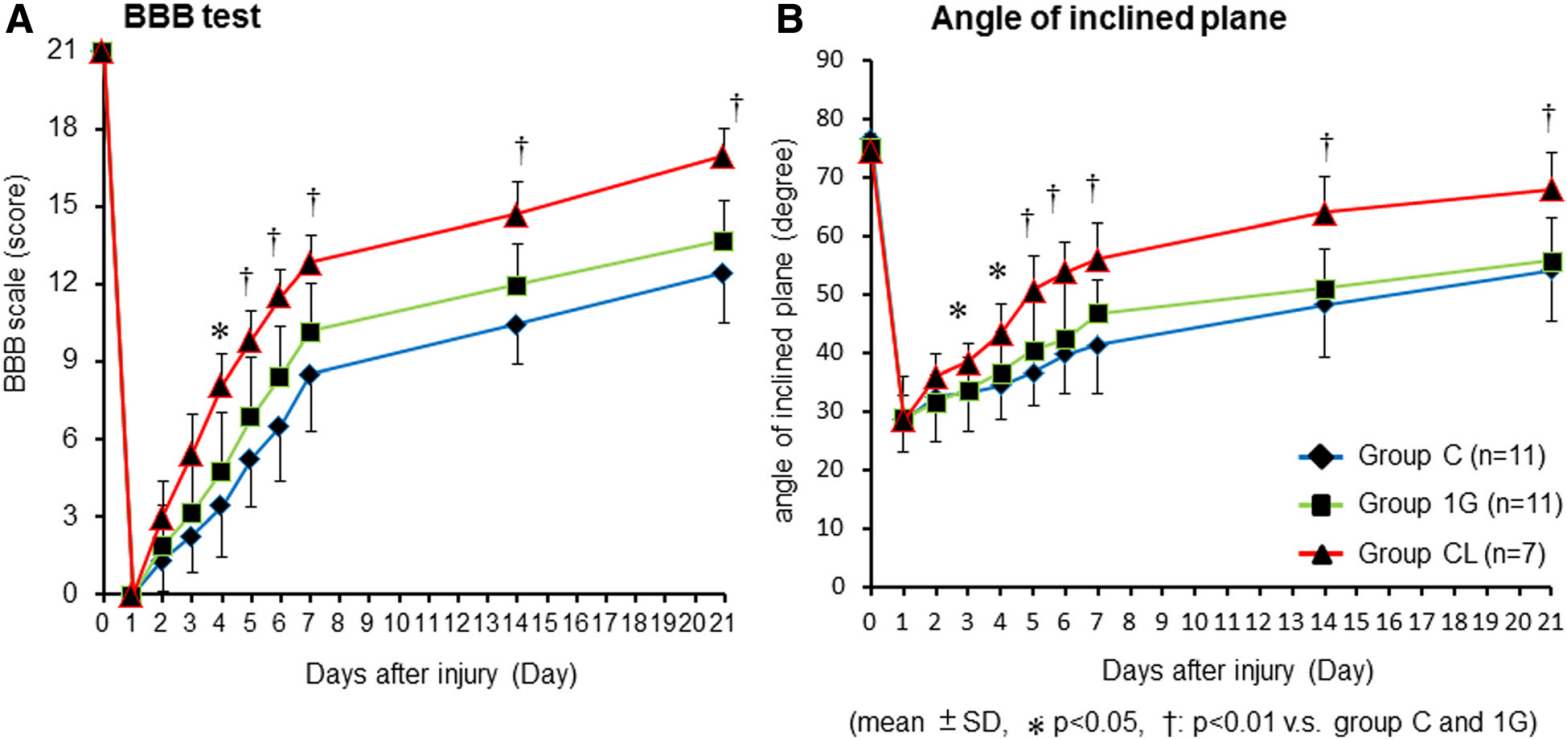
**Locomotor recovery.** These graphs show the analysis of locomotor recovery as measured by the BBB score and the inclined-plane task score from the day on which SCI was induced (day 0) to 21 days later. (**A**) BBB scores were significantly higher in group CL than in group 1G from day 5 to day 21 after SCI (*P* value < 0.01). (**B**) The angle of the inclined plane was also significantly greater in group CL from day 5 to day 21 (*P* value < 0.01).

In total, 29 adult female Fischer/F344 rats received a standardized contusion of the spinal cord (11 each in Control and 1G groups, seven in group CL). Dropping a weight with a force of 50 g/cm resulted in complete paraparesis, and rBMSCs of either group were transplanted immediately after the crush injury. For 21 days after the crush injury, the BBB and inclined-plane task scores gradually improved in all rats (Figure 3A, B). Group CL showed the greatest improvement in motor function from 5 to 21 days after SCI, as compared with the controls and group 1G. The difference in the BBB and inclined-plane task scores between group CL and group 1G reached statistical significance by 5 days after the injury.

**Figure 4:**
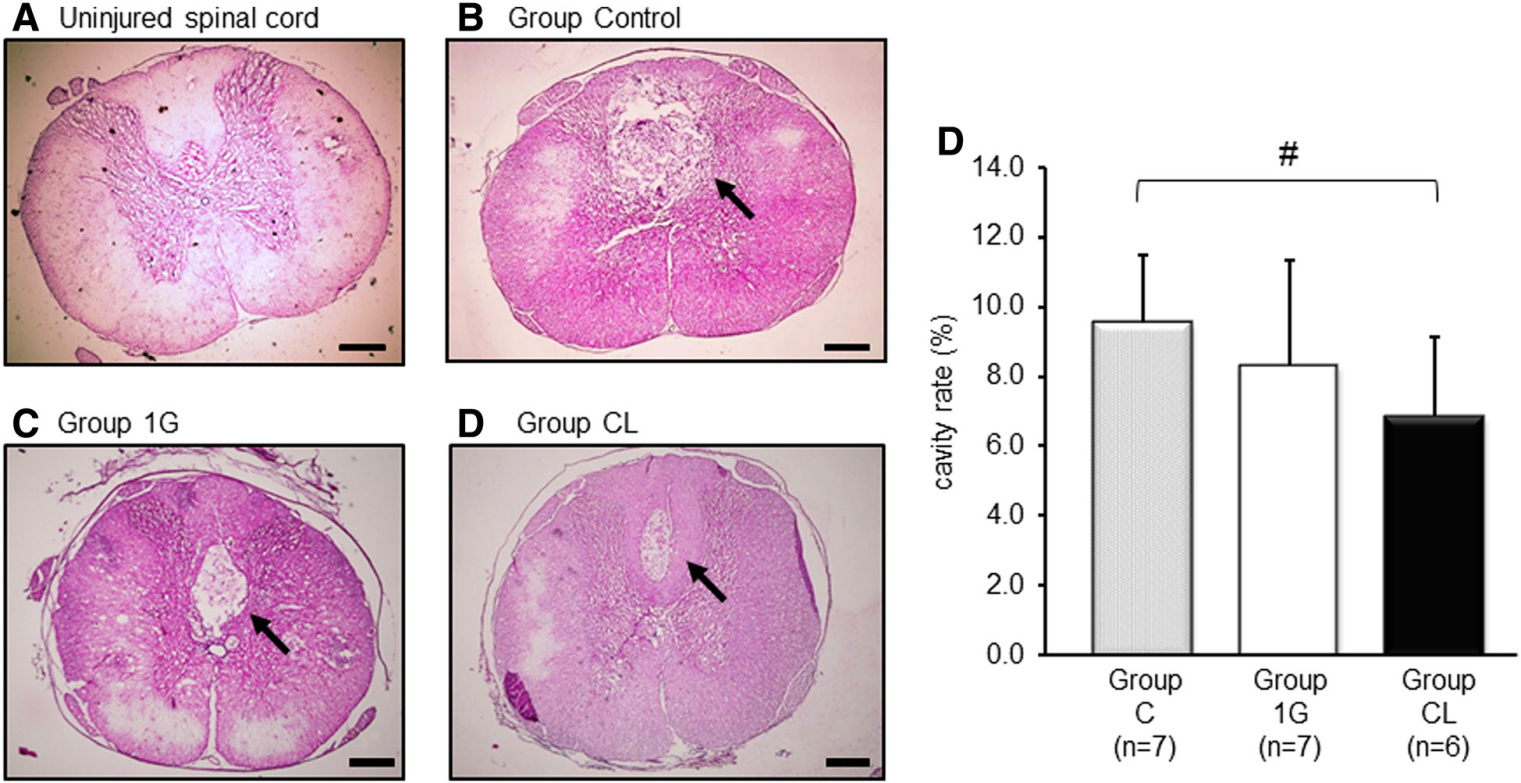
**Cavity formation after SCI.** Cavity formation after SCI stained with hematoxylin and eosin (**A**) rat uninjured spinal cord, (**B**) group Control, (**C**) group 1G and (**D**) group CL). Scale bars, 300 μm. The cavity formations (arrows) were markedly reduced in the groups that had received rBMSC transplants. Only very small cavities were identified within the SCI lesions in group CL. The graph (**D**) shows the difference in the volume of cavities at 21 days after SCI among group Control (*n* = 7), group 1G (*n* = 7), and group CL (*n* = 6). Data are expressed as mean ± SD. The values of the cavity size were significantly different between group CL and group Control (*P* value < 0.05). Group C, *n* = 11; Group 1G, *n* = 11; Group CL, *n* = 7.

The cavity formations, as assessed by H&E staining of coronal sections at 21 days after SCI, were markedly reduced in the groups that had received rBMSC transplants (Figure 4A-C). Only very small cavities were identified within the SCI lesions in group CL; the cavity ratio of group CL was also significantly reduced compared with that of group Control (*P* < 0.05; Figure 4D).

**Figure 5:**
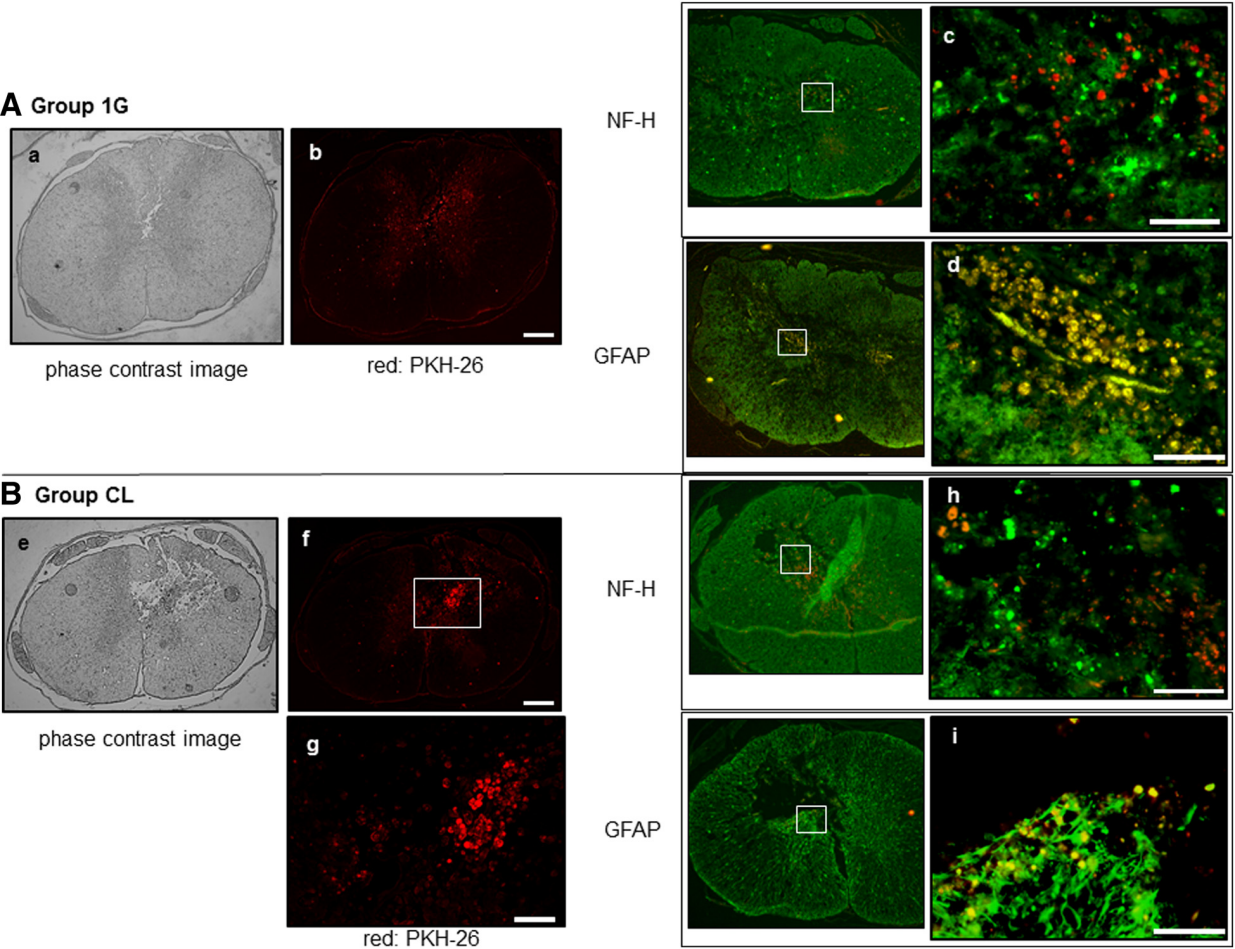
**Immunostained images of each group at 21 days after transplantation.** Phase-contrast microscopic images of spinal cord from each group (**a**, **e**), and transplanted cells labeled with PKH-26 (**b, f, g**) are shown. In group CL, clustering of the migrated PKH-26-positive cells was observed around the SCI lesion (**f**, **g**). Immunostained images of each group are shown © NF-H in group 1G, (**d**) GFAP in group 1G, (**h**) NF-H in group CL, (**i**) GFAP in group CL). Green staining indicates cells positive for the differentiation marker. Yellow-staining cells indicate grafted cells expressing each neural marker. Transplanted cells had a tendency to express higher levels of GFAP than NF-H in group 1G and in group CL (**c, d, h, i**). Scale bars, 300 μm (**b** and **f**), and 100 μm (**c**, **d**, **g**, **h,** and **i**).

On evaluation at 21 days after SCI, many transplanted rBMSCs, labeled with PKH-26, had migrated into the SCI lesions; the number of PKH-positive cells was noticeably higher in group CL than in group 1G (Figure 5A, B) (see Additional file 3: Figure S2: the phase-contrast images of rat uninjured spinal cord and contusional spinal cord without cell transplantation). Furthermore, clustering of the migrated cells was observed in the SCI lesion in group CL (Figure 5B).

### Effects of cell transplantation in SCI model rats: immunohistochemical analysis

Transplanted cells showed higher expression of GFAP than of NF-H in both groups 1G and CL. No statistically significant difference was noted in the number of GFAP-positive cells between the 1G and CL groups (Figure 5A, B).

**Figure 6:**
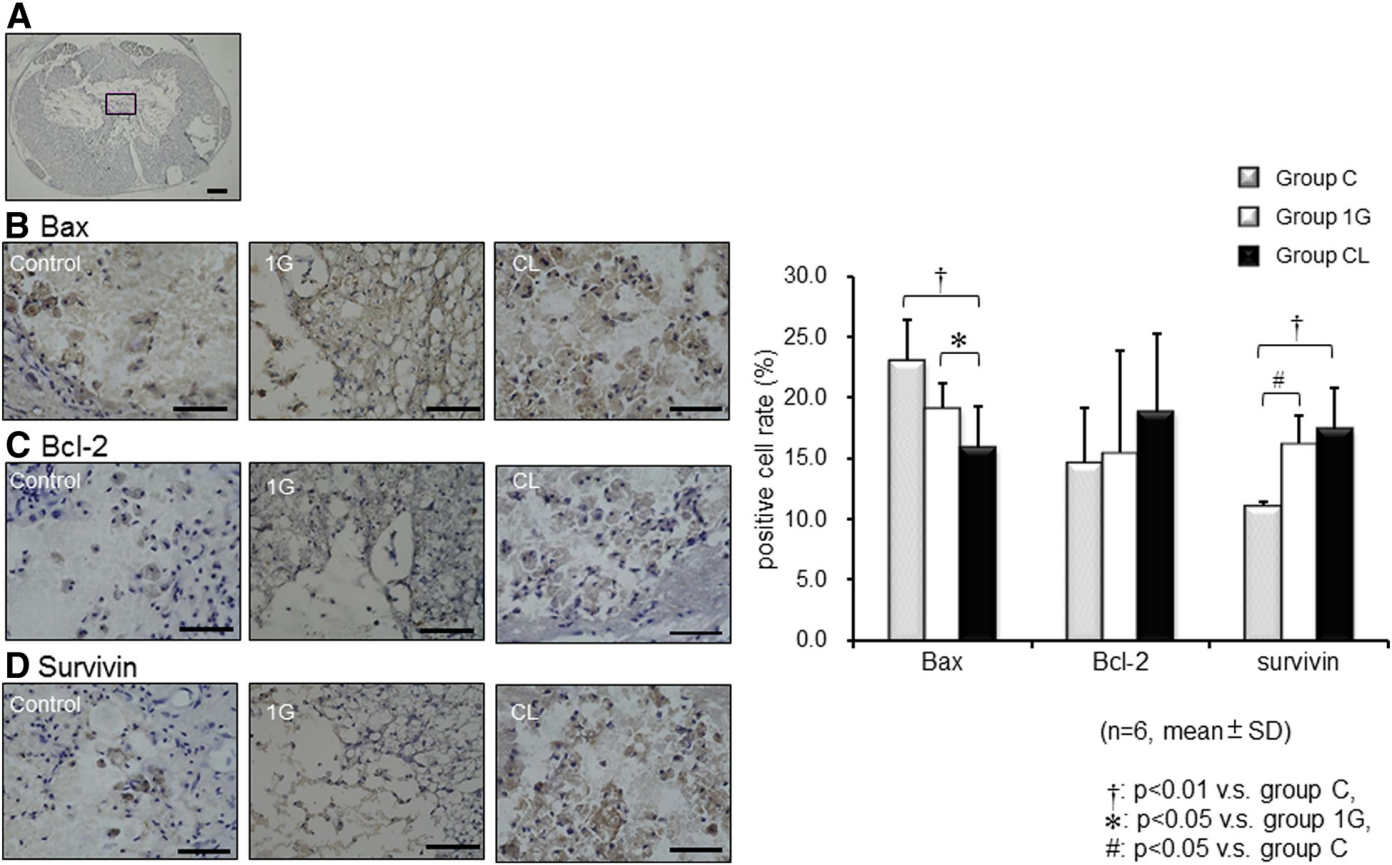
**Expression of apoptosis markers.** Frozen sections were stained with apoptosis markers, Bax (**B**), Bcl-2 (**C**), and survivin (**D**), to examine the neurotrophic effects activated by grafted cells against apoptosis. The graph shows the positive cell percentage around SCI lesion in the frozen spinal cord sections (**A**) of each group (*n* = 6 per group). The number of Bax-positive cells was significantly decreased in group CL rats compared with the control group (*P* value < 0.01) and group 1G (*P* value < 0.05). Moreover, the number of survivin-positive cells was significantly increased in groups 1G and CL compared with the control (*P* value < 0.01; *P* value < 0.05). Bcl-2 expression was not statistically significantly different between groups. Data are expressed as mean ± SD. Scale bars, 300 μm (**A**), 100 μm (**B**, **C**, and **D**).

Expression of the apoptosis markers Bax2, Bcl-2, and survivin were examined in the frozen sections of each group on day 21 (Figure 6). The positive cell percentage for the apoptosis-promoting factor Bax was significantly decreased in group CL compared with group Control and group 1G. Moreover, the expression of the apoptosis-inhibitory factor survivin was significantly increased in group 1G and CL compared with the control. Bcl-2 expression was not significantly different among the groups.

Taken together, the findings indicate that transplantation of the rBMSCs in group CL contributed to a significant improvement of motor function and morphologic features compared with the improvement observed in group 1G.

## Discussion

We report here the remarkable neuroregenerative effect of rBMSCs cultured in simulated microgravity on functional recovery after SCI; to our knowledge, this is the first study in which rBMSCs cultured in simulated microgravity were transplanted into an SCI model.

Previously, BMSCs have been reported to exhibit distinct effects on the recovery of injured spinal cords and to improve locomotion in SCI rats; moreover, cavity formation in the spinal cord was reported to be reduced when BMSCs were infused into the cerebrospinal fluid with acute SCI [24,25]. To examine possible future clinical applications of cells grown in simulated microgravity, we used a spinal-contusion model involving a weight-dropping method. In this study, grafted rBMSCs promoted functional improvement, which was accompanied by the suppression of cavity formation 3 weeks after SCI. As previously reported [13], functional recovery of the spinal-contusion models by using a weight-dropping method was less prominent and reached a plateau during the third week after SCI. In this study, we transplanted cells immediately after a surgical procedure to examine the therapeutic benefits of the transplanted cells concerning the multiple pathogenic signals that function synergistically during the early phase after SCI. The present study confirmed possibilities that rBMSCs cultured under simulated microgravity have the pluripotency and the advantage as grafting cells for the early phase of SCI repair.

Previous studies have shown that microgravity suppresses the differentiation of human osteoblast cells [15], human hematopoietic progenitor cells [18], and rat myoblasts [16]. Simulated microgravity also allows novel culture methods for mouse ES cells that do not require leukemia inhibitory factor or animal-derived supplements [14]. The 3D-clinostat is a device that allows generation of a multidirectional *g* force, resulting in an environment with an average of 10^-3^*g*[14,16]. Our previous study showed that cells cultured in a 3D-clinostat exhibit suppressed cell differentiation [16,17,26] and induction of growth inhibition through reduction of mitochondrial activity [17]; moreover, simulated microgravity enhanced the chemosensitivity of malignant glioma cells [27].

In the present study, simulated microgravity was considered to induce growth inhibition and to maintain the undifferentiated state of rBMSCs, because the rBMSCs cultured under simulated microgravity were smaller and had a dome-like shape, and the expression of *Oct-4* mRNA was observed only in group CL cells.

A recent study showed that when undifferentiated ES cells are transplanted, they act in a neuroprotective manner and exert antinociceptive and therapeutic effects after excitotoxic SCI [28]. Another study showed that transplantation of murine ES cells that had been undifferentiated into GABAergic neurons significantly induced recovery of sensorimotor function after traumatic brain injury, whereas animals transplanted with astrocytes did not show any recovery [29]. Several studies have shown the superiority of using undifferentiated stem cells for transplantation after SCI for facilitating functional recovery [29-31]; the results of the present study were consistent with the findings of other transplantation studies, because simulated microgravity induced an undifferentiated state in the grafted rBMSCs.

By using the specific SDF-1 receptor (CXCR4), we found significant expression of the chemokine stromal-cell-derived factor-1 (SDF-1) in rBMSCs cultured under simulated microgravity. The interaction of SDF-1 with CXCR4 mediates the homing of hematopoietic stem cells to the bone marrow [32]; SDF-1 is also known to induce migration of neural cells [30,33]. In this study, the number of transplanted rBMSCs that had migrated into the SCI lesion was markedly greater in group CL and the cavity formation after SCI was markedly reduced in group CL compared with group 1G. Our results suggest that simulated microgravity mediated the extended expression of CXCR4 in the rBMSCs, and that the interaction of SDF-1 with CXCR4 then facilitated the trafficking of rBMSCs to the SCI site.

Several studies have shown that BMSCs differentiate into neural cells, including astrocytes and neurons [6,34,35]; conversely, other studies have indicated that transplanted BMSCs do not differentiate into neural cells in the spinal cord [12,20]. Our previous study, by using a mouse model of cerebral contusion, showed that the efficacy of grafting mouse BMSCs was attributed not only to the cells differentiating into neuronal cells but also to the factors released by grafted cells that suppressed the formation of a glial scar and that enhanced the elongation of axons [17]. It has been suggested that transplanted BMSCs may not survive long enough to be integrated into the spinal cord tissue [12,36] and that BMSCs, despite their limited survival time, enhance tissue matrix formation and axonal outgrowth in SCI by releasing diffusible neuroprotective factors [6,25,30,34,37]. This probably contributed to the distinct improvement of locomotor behavior as well as the reduction of the cavity formation noted in our study. Although, in our study, the transplanted rBMSCs expressed the differentiation marker GFAP, it is unlikely that the efficacy of rBMSCs for improving the injured spinal cord relates to these cells forming spinal cord tissue, because of the small number of rBMSCs used for transplantation.

It has been shown that stem cell transplantation can improve neurologic function by several mechanisms, some of which are neuroprotective effects on host neurons from trophic factors secreted by transplanted cells, and the reestablishment of functional neural networks through the integration of transplanted cells [38,39]. All things considered, the beneficial effect may rather relate to the release of neuroprotective factors by the BMSCs [40-42].

Neurotrophins are a family of proteins that are best characterized by their modulation of survival, differentiation, and apoptosis of cells in the nervous system, and exogenously applied trophic factors, including BDNF, IGF-1, NT-3, GDNF, and HGF, have been shown to be effective for SCI. Although the trophic factors derived from BMSCs promote neurite extension and survival *in vitro*, their roles in the functional recovery of SCI are still largely unknown. In this study, we investigated the expression of *NGF* and *BDNF* mRNAs in graft cells, but found no difference between groups 1G and CL with regard to the expression of these mRNAs. The mRNA expressions of NGF and BDNF are only by a semiquantitative method. Therefore, to investigate the trophic effect of the grafted rBMSCs, we analyzed apoptosis markers in the SCI lesions, because SCI induces a series of endogenous biochemical changes that lead to secondary degeneration, including apoptosis. Mitochondrial apoptosis mediated by p53 is likely to be an important mechanism of cell death in SCI. Expression of p53 was observed in neurons, oligodendrocytes, and astrocytes after SCI, and upregulation of phospho-p53 and Bax, and downregulation of Bcl2, were detected after SCI [43]. In this study, the fact that the expression of the apoptosis-promoting factor Bax significantly decreased and that of the apoptosis-inhibitory factor survivin significantly increased in group 1G and CL rats compared with group Control, demonstrated the trophic, antiapoptotic effect of the grafted rBMSCs. Apoptosis and free-radical damage are the prominent processes involved in secondary degeneration after SCI [43,44]. Our results suggest that the grafted BMSCs immediately after injury prevented the secondary degeneration and enhanced the proliferation of the axons to a greater extent in group CL rather than in group 1G.

## Conclusion

rBMSCs cultured under simulated microgravity suggested to be induced undifferentiated state and migration ability. rBMSCs cultured under simulated microgravity had greater trophic effects on reestablishment and survival of host spinal neural tissues than those cultured under 1g, and contributed to enhanced functional improvement after SCI *in vivo*.

## Abbreviations

Bax: B-cell CLL/lymphoma 2-associated X protein; BBB scale: Basso-Beattie-Bresnahan locomotor rating scale; Bcl-2: B-cell CLL/lymphoma 2; BDNF: brain-derived neurotrophic factor; BMSC: bone marrow stromal cell; CXCR4: CXC-chemokine receptor 4; ES cell: embryonic stem cell; GFAP: glial fibrillary acidic protein; G3PDH: glyceraldehyde-3-phosphate dehydrogenase; GDNF: glial cell line-derived neurotrophic factor; HGF: hepatocyte growth factor; H&E: hematoxylin and eosin; IGF-1: insulin-like growth factor-1; NF-H: neurofilament heavy chain; NGF: nerve growth factor; NT-3: neurotrophin-3; Oct-4: octamer-binding transcription factor 4; PBS: phosphate-buffered saline; rBMSC: rat BMSC; RT-PCR: reverse transcription polymerase chain reaction; SCI: spinal cord injury; SDF-1: stromal-cell-derived factor-1.

## Competing interests

The authors have no personal financial or institutional interest in any of drugs, materials, or devices described in this article.

## Authors’ contributions

TM participated in the design of the study, carried out the experiments of the *in vitro* and *in vivo* study, data analysis and interpretation, and manuscript writing. MT conceived of the study, participated in its design and coordination, carried out the *in vitro* and *in vivo* study, data analysis, and interpretation, and final approval of manuscript. SY participated in the design of the study, data analysis and interpretation, and financial support. TM participated in the design of the study, carried out the *in vitro* and *in vivo* experiments, data analysis and interpretation, and performed the statistical analysis. MM participated in the design of the study, carried out the experiments *in vitro* and *in vivo,* and data analysis and interpretation. YK participated in the design of the study, data analysis, and interpretation. LY conceived of the study, participated in its design and coordination, data analysis, and interpretation, final approval of the manuscript, and financial support. KK participated in its design and coordination, data analysis and interpretation, helped to draft, and gave final approval of the manuscript and financial support. All authors read and approved the final manuscript for publication.

## Supplementary Material

Additional file 1: Table S1 Polymerase chain reaction primers and conditions. Click here for file

Additional file 2: Figure S1 The morphologic changes of rBMSCs cultured under microgravity. On Day 4, the cells became smaller and rounder. On Day 7, the cells became much smaller and dome-like in shape. Scale bars, 100 μm. Click here for file

Additional file 3: Figure S2 The phase-contrast images of rat spinal cord. (A) Rat uninjured spinal cord. (B) Rat contusional spinal cord without cell transplantation. Scale bars, 300 μm. Click here for file
