# Supplementary material for: Simulated microgravity facilitates cell migration and neuroprotection after bone marrow stromal cell transplantation in spinal cord injury
Source: Stem Cell Res Ther. 2013 Apr 1;4(2):35. doi: 10.1186/scrt184 (PMC3706926; doi:10.1186/scrt184)
Supplement: Additional file 1: Table S1 — Polymerase chain reaction primers and conditions. [file scrt184-S1.doc]

| **Gene name** | **Primer sequence** | **RT-PCR condition** | **Product size** |
| --- | --- | --- | --- |
| **Oct-4** | **F：5’-AAGCTGCTGAAACAGAAGAGG-3'**  **R：5’-ACACGGTTCTCAATGCTAGTC-3'** | **95℃30s, 58℃30s, 68℃45s, 35cycles** | **272 bp** |
| **CXCR4** | **F：5’-TTCTCATCCTGGCCTTCATC-3’**  **R：5’-TGGAGTGTGACAGCTTGGAG-3’** | **95℃30s, 58℃30s, 68℃60s, 37cycles** | **318 bp** |
| **NGF** | **F:5’-GCCCACTGGACTAAACTTCAGC-3’**  **R:5’-CCGTGGCTGTGGTCTTATCTC-3’** | **95℃30s, 55℃30s, 68℃60s, 35cycles** | **349 bp** |
| **BDNF** | **F:5’-GGTCACAGTCCTGGAGAAAG-3’**  **R:5’- GTCTATCCTTATGAACCGCC-3’** | **95℃30s, 55℃30s, 68℃60s, 35cycles** | **214 bp** |
| **G3PDH** | **F：5’-TCTTCACCACCATGGAGAAGGCTG-3’**  **R：5’-ACAGTCTTCTGAGTGGCAGTGATG-3’** | **95℃30s, 60℃45s, 68℃60s, 25cycles** | **262 bp** |

Primers and amplification conditions used for PCR (Forward primers: F, reverse primers: R).
