# Supplementary figures and images for: Simulated microgravity facilitates cell migration and neuroprotection after bone marrow stromal cell transplantation in spinal cord injury
Source: Stem Cell Res Ther. 2013 Apr 1;4(2):35. doi: 10.1186/scrt184 (PMC3706926; doi:10.1186/scrt184)

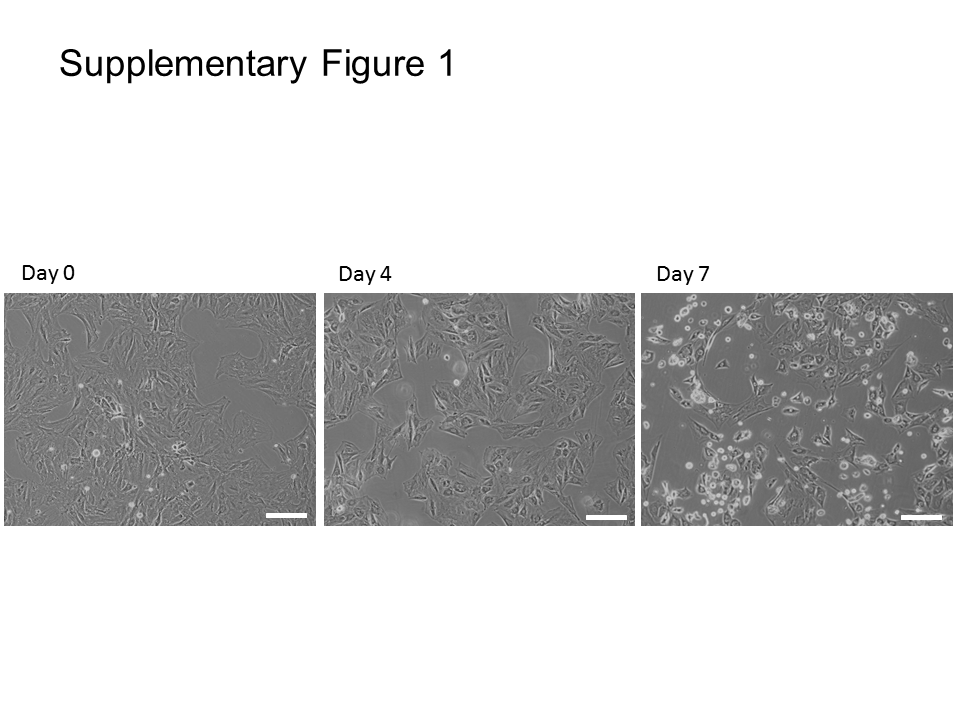

Supplement: Additional file 2: Figure S1 — The morphologic changes of rBMSCs cultured under microgravity. On Day 4, the cells became smaller and rounder. On Day 7, the cells became much smaller and dome-like in shape. Scale bars, 100 μm. [file scrt184-S2.tiff]

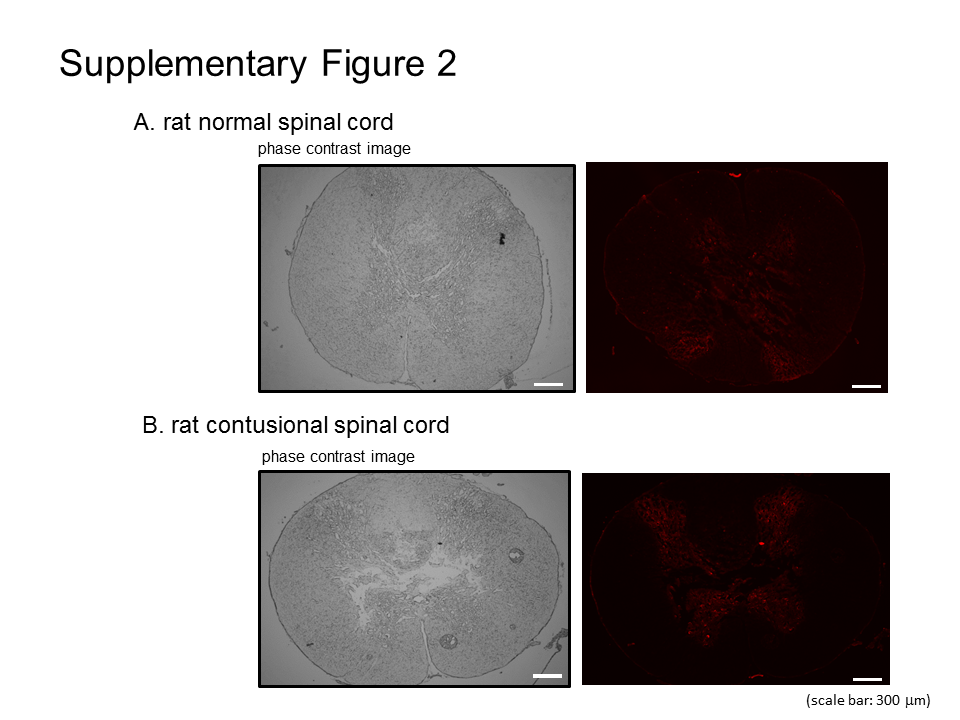

Supplement: Additional file 3: Figure S2 — The phase-contrast images of rat spinal cord. (A) Rat uninjured spinal cord. (B) Rat contusional spinal cord without cell transplantation. Scale bars, 300 μm. [file scrt184-S3.tiff]
